# The Role of Enamel Proteins in Protecting Mature Human Enamel Against Acidic Environments: A Double Layer Force Spectroscopy Study

**DOI:** 10.1007/s13758-011-0014-6

**Published:** 2012-02-17

**Authors:** Gennady V. Lubarsky, Raechelle A. D’Sa, Sanjukta Deb, Brian J. Meenan, Patrick Lemoine

**Affiliations:** 1 Nanotechnology and Integrated Bioengineering Centre (NIBEC), School of Engineering, University of Ulster, Shore Road, Newtownabbey, Co., Antrim, BT37 0QB Northern Ireland, UK; 2 Department of Biomaterials, King’s College London Dental Institute, London, SE1 9RT UK

## Abstract

Characterisation of the electrostatic properties of dental enamel is important for understanding the interfacial processes that occur on a tooth surface and how these relate to the natural ability of our teeth to withstand chemical attack from the acids in many soft drinks. Whereas, the role of the mineral component of the tooth enamel in providing this resistance to acid erosion has been studied extensively, the influence of proteins that are also present within the structure is not well understood. In this paper, we report for the first time the use of double-layer force spectroscopy to directly measure electrostatic forces on as received and hydrazine-treated (deproteinated) enamel surfaces in solutions with different pH to determine how the enamel proteins influence acid erosion surface potential and surface charge of human dental enamel. The deproteination of the treated samples was confirmed by the loss of the amide bands (~1,300–1,700 cm^−1^) in the FTIR spectrum of the sample. The force characteristics observed were found to agree with the theory of electrical double layer interaction under the assumption of constant potential and allowed the surface charge per unit area to be determined for the two enamel surfaces. The values and, importantly, the sign of these adsorbed surface charges indicates that the protein content of dental enamel contributes significantly to the electrostatic double layer formation near the tooth surface and in doing so can buffer the apatite crystals against acid attack. Moreover, the electrostatic interactions within this layer are a driving factor for the mineral transfer from the tooth surface and the initial salivary pellicle formation.

## Introduction

Dental enamel is the outermost protective shell of our teeth and is in a constant state of demineralization and remineralization, depending on the acidity of the dental environment. A reduction of the local pH at the enamel surface can ensue following an intake of fizzy drinks or acidic foods or via the metabolism of oral bacteria. Under normal conditions, any damage to hydroxyapatite (HA), the main mineral constituent of dental enamel, is quickly restored by minerals and enzymes from saliva. However, even small alterations in this equilibrium may lead to demineralization [1] and softening [2] of enamel producing dental erosion and creating weak spots for decay to proceed into the tooth. Ionic regulation of tooth enamel is a complex process that includes diffusion of ions into and out of enamel; these ions are transported through dentin and also pellicle and dental plaque formation. In several models proposed for dental carries progression, it has been shown that these electrostatically driven processes are major factors to be considered in reducing tooth decay [3–5]. Previously, the potential drop over enamel membranes has been measured using the microwells technique on as received and carious human enamel [6] and during caries attack [7]. It has also been shown that the effect of permselectivity in enamel is a major driving force for ions transport through enamel.

When dental enamel is placed in contact with saliva or a beverage, ions accumulate on its surface forming a surface charge layer [8]. Since these interactions occur in aqueous solutions an electrostatic double layer will exist either through the dissociation of surface functional groups and/or through the adsorption of ions from solution. In this case, the surface charge is balanced by the accumulation of an equal number of oppositely charged counterions that are either bound to the surface to form the Stern layer or present in the liquid media that occurs above the surface to form an electrostatic double layer. Since salivary enzymes and proteins are usually charged molecules in aqueous solution, the presence of this electrostatic double layer with its associated electric field will have an important influence on the interaction between enamel surface and the adsorbed molecules. Selective binding of these molecules is an initial stage of formation of a dental pellicle—a natural protection layer deposited on the enamel surface preventing continuous deposition of salivary calcium phosphate.

**Fig. 1:**
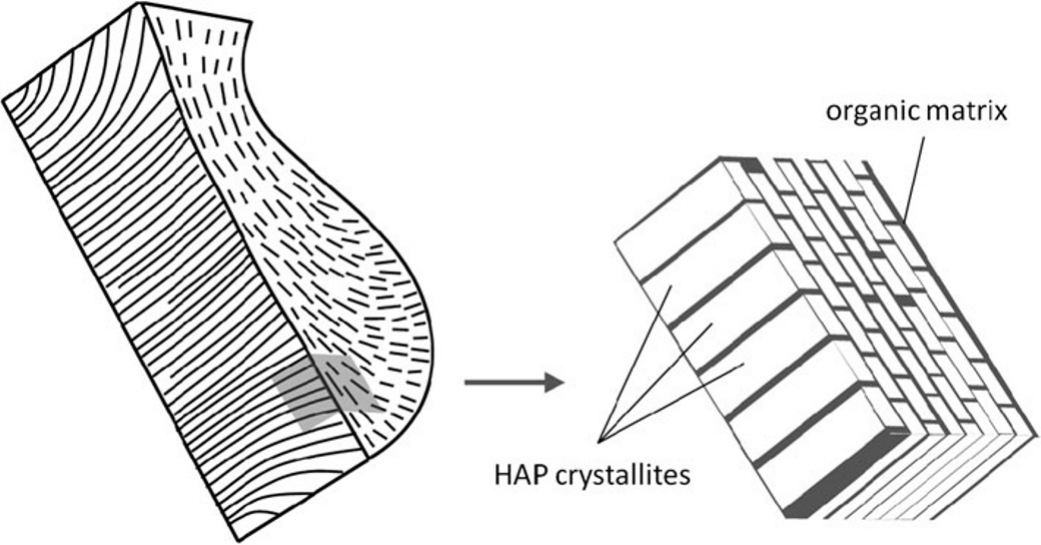
Schematic illustration of crystal orientation of hydroxyapatite crystallites in enamel keyhole-shape rod unit showing the mineral and organic matrix composite structure

On the nanoscale, enamel is a complex matrix of minerals and proteins (Fig. 1). This matrix consists of highly organized array of very fine HA crystals embedded in a protein-rich sheath [9]. Various hydroxyapatite substrates have been extensively studied as model dental enamels. The electrostatic properties of HA pellet surfaces have been investigated with spatially resolved specific force spectroscopy using nanosized probe functionalized tips [10]. The adhesion force has been measured between hydroxylated or carboxylated cantilever tips and HA crystals obtained from maturation stage enamel between pH 2 and 10 [9, 11]. Most recently, the colloidal probe atomic force microscopy method has been applied to investigate the surface properties of hydroxyapatite surfaces [12].

The organic content of dental enamel consists mainly of proteins: amelogenin, enamelin, and ameloblastin [13]. Although this fraction is small, ~1 wt% [14], it is very finely dispersed through the hierarchical structure of enamel; it binds together nanoscale HA crystals both within the rods and the sheath of enamel. It is this property that makes dental enamel permeable to ions allowing the delivery of mineral into the bulk of enamel and thereby allowing for regulation of crystal surface charges. The majority of studies of the mechanisms of damage to dental enamel have considered the direct effects on the HA component to be the most significant factor. However, the ability of protein molecules to acquire and alter the net charge according to the surrounding pH [15] is an important factor in this respect, in that this organic fraction may contribute to processes of teeth surface charge regulation, protection and remineralisation.

Hence, the aim of this research is to study the influence of matrix proteins on the surface properties of dental enamel. The relatively new technique of double-layer force spectroscopy (DLFS) with three-electrode electrochemical control has been used to measure the charge condition on both as received and deproteinated enamel surfaces in solutions with different values of pH. To the best of the authors’ knowledge, this is the first time that this measurement technique has been applied to the study of human dental enamel. The experimental setup allows for the direct measurement of piconewton (pN) level forces in fluid between a nanosized probe tip with fixed potential and the charge generated as a function of separation distance from a sample of interest [16]. By comparing DLFS data on approach of the probe tip to the sample surface to the nonlinear Poisson–Boltzmann-based electrostatic double layer theory [17, 18], an estimation of the surface charge per unit area, *σ* (C/m^2^) can be made.

In this work, enamel deproteination was performed by a procedure initially proposed by Termine et al. [19] where hydrazine was used to eliminate the organic matrix from compact bones. Recently, the possibility of chemical and physical alterations caused by the hydrazine deproteination process to bone samples has been extensively studied [20]. The results obtained clearly show that the hydrazine process does not alter bone mineral composition or morphology and is regarded as one of the most effective deproteination processes available [21–24].

## Materials and Methods

### Artificial Saliva

The aim of present study is to investigate the contribution of proteins present within the bulk enamel to its electrostatic properties. We opted for a saliva model system without organic content, a Artificial Saliva Gal Fovet (SAGF) media to eliminate the contribution from salivary proteins. In addition, the SAGF is a translucent and non-viscous solution with low oxidation currents and suitable for DLFS experiments. SAGF was prepared accordingly to the process described by Gal et al. [25] with the composition presented in Table 1. The pH of the resulting solution was verified by microprocessor-based pH meter (Hanna Instruments, Leighton Buzzard, UK) to be 6.8. The pH of the solution was varied by adding citric acid (Sigma-Aldrich Company, Dorset, UK) to the SAGF media to adjust the pH to 4.2.

**Table 1:** Composition of SAGF medium (from Ref. [25])

Compound	Concentration (mg l^−1^)	Compound	Concentration (mg l^−1^)
NaCl	125.6	Urea	200.0
KCl	963.9	Na_2_SO_4_, 10H_2_O	763.2
KSCN	189.2	NH_4_Cl	178.0
KH2PO4	654.5	CaCl_2_, 2H_2_O	227.8
Urea	200.0	NaHCO_3_	630.8

### Samples Preparation

Human dental enamel specimens of dimensions approximately, 5 × 5 × 2 mm, were prepared from as received, caries-free regions of a second molar extracted during dental treatment. The samples were obtained under the procedures operating prior to the establishment of the UK Human Tissue Act (2008). The samples were sectioned using a water-cooled diamond-tipped annular saw (Ameritool 4, Manchester Minerals, and Stockport, UK). The enamel blocks were ground using 1,200-grit silicon carbide paper under low flow of SAGF solution to remove the very outer enamel so as to provide a flat surface. The specimens were then ultrasonicated in SAGF solution for approximately 5 min at room temperature to remove polishing debris. The final fine polishing was carried out using 0.3 μm and 0.05 colloidal Al_2_O_3_ to achieve a mirror finish and ultrasonicated again.

In order to remove the enamel proteins, samples were immersed directly into 99.9% hydrazine hydrate (Cat. No. 225819, Sigma-Aldrich Company, Dorset, UK) in a 15 ml polypropylene flask and sealed. The flask was placed into a thermostat at 70°C for 2 weeks, to accelerate the deproteination process. After this step, the reagent was decanted and the sample was washed successively in ethanol/water solutions with ethanol concentration of 50, 75 and 100%. This sample was dried in open air and immersed into the SAGF solution. Prior to analysis, the variously prepared enamel specimens were stored in SAGF solution for about 24 h to reach stable hydration conditions and avoid drift during the force spectroscopy experiments.

### FTIR-Spectroscopy

The enamel samples were characterized before and after the deproteination process using a Varian 640-IR (Agilent Technologies, Santa Clara, CA, USA) infrared spectrometer. The spectra were acquired at a resolution of 4 cm^−1^ for 128 scans from 400 and 4,000 cm^−1^ using a diffuse reflectance attachment. The Kramers–Kronig transformation [26] was used to convert the spectra to a form that does not contain any derivative peak shapes. The reference spectrum was acquired on the aluminium sample holder.

### Atomic Force Microscopy

Force spectroscopy was carried out in a commercial scanning probe microscope (PicoSPM equipped with PicoScan 2100 controlling units, Molecular Imaging, USA). The root-mean square (RMS) roughness of the finished surface was obtained from topography images measured by AFM. All experiments were performed in a liquid cell filled with SAGF solution. The SAGF solution was injected into the cell, using a syringe and allowed to equilibrate for 1 h to allow for adsorption within the enamel surfaces. The images were subjected to first order flattening to remove offset and tilt of each line and the RMS roughness values were calculated using the software provided by the WSxM software [26].

**Fig. 2:**
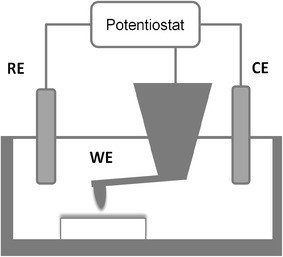
Schematic drawing of the experimental three-electrode set up

A PicoAFM small range scanner operating with a closed loop in*z* direction was used in spectroscopic mode. A three-electrode system (Fig. 2) was installed in the liquid cell. The working electrode (WE) was a 450 μm long rectangular silicon cantilever with a conductive platinum/iridium coating (SCM-PIC, Veeco). The counter electrode (CE) and reference electrode (RE) were produced from silver wire and immersed into the cell. The cantilever and the electrodes were electrically linked to the potentiostat (PicoStat, Molecular Imaging) which kept the potential, ψ_p_, between the tip and the reference electrode at a constant controlled value. As the sample was electrically insulated in the liquid cell, its surface potential was kept constant during force measurement. The electrolyte was hydrodynamically static during the measurements.

The force spectroscopy experiment consists of a spherical tip mounted on the end of a force-sensing lever interacting with a planar surface. Prior to measurement, the normal spring constant were measured for each cantilever using the method proposed by Sader et al. [27]. The method measured the length and width of the cantilever, as well as the resonant frequency and quality factor of the resonant peaks. The measured spring constant of the cantilevers used in the DLFS experiments was 0.035 ± 0.008 Nm^−1^.

The AFM tip radius was calculated from a sphere fit to the images obtained from a standard AFM calibration method (Model No. P-000-0004-0 Pacific Nanotechnology, CA, USA) using SPIP software (Image Metrology A/S, Denmark). The effective radius of the tip was determined to be 58 ± 7 nm.

Each set of data was an average of ten approach and retraction cycles. The data were obtained by cycling the z-piezo over a specific distance of 300 nm at a frequency of 0.5 Hz with a typical scan size of 1 μm. The force-response of the lever is described by Hooke’s Law *F* = −*k*Δ_*C*_, where *k* is the spring constant corresponding to normal deflection Δ_*C*_, of the lever. The deflection of the cantilever (∆_*C*_) was recorded as a function of piezo movement (*z*) during both approach and retraction of the tip. Resulting plots revealed two distinct regions, 1/a flat portion representative of the cantilever in its rest position (Δ_*C*_ = 0) not in contact with the surface and 2/a sloped region, indicative of a linear cantilever deflection with respect to the sample displacement (Δ_*C*_ = *z*) representing points where the tip is in contact with the sample. Force-distance curves were subsequently obtained by converting the deflection data and accounting for the relative tip-sample distance, as described by Ducker et al*.* [28] and using the SPIP software package. Practically, the cantilever sensitivity (V/nm) was automatically calculated from the slope of the retracting curve. The cantilever deflection was calculated by dividing the deflection voltage with the cantilever sensitivity. The separation between sample and cantilever was calculated from sum of deflection and height signals. The found minimum separation was set to zero The portion of the force distance curve that deviates from the cantilever rest fitted line to where it meets the zero tip-surface separation position was used to fit the experimental data to the theoretical model using a least-square fitting.

## Results and Discussion

### Topography Mapping

**Fig. 3:**
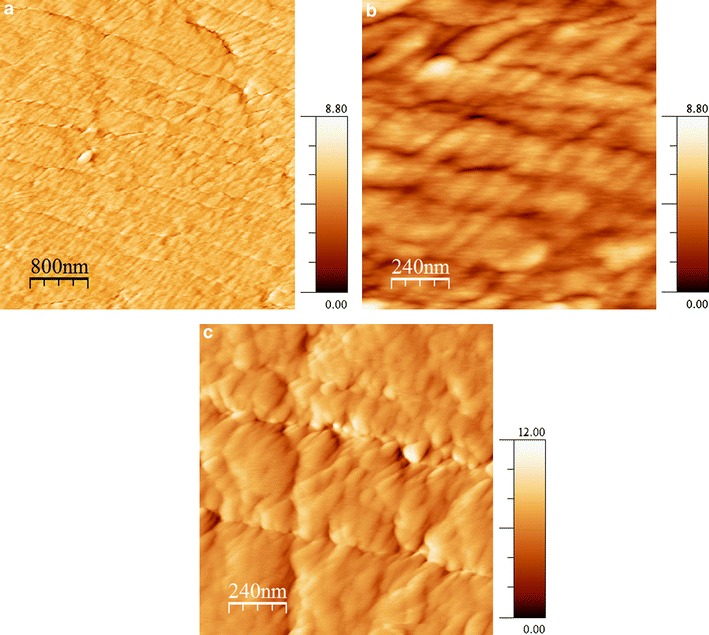
Topography images of deproteinated (**a**, **b**) and as received (**c**) enamel surfaces in SAGF solution with pH 6.8 obtained by deflection contact mode AFM

The surface morphology of the dental enamel samples were studied using AFM contact mode topographical imaging in SAGF solution at pH 6.8. Typical images of the as received and deproteinated enamel surfaces are given in Fig. 3. The measured RMS roughness of both samples is about 10 nm.

A topography image of the deproteinated surface indicates clearly that the nanostructure of human enamel has been exposed and consists of very fine HA crystallites aligned in the direction perpendicular to the surface normal. These crystallites are prismatic in cross section, with mean dimensions of 68 × 26 nm, values that are commensurate with those reported in other published work [29]. The separation between crystallites, ~10 nm in Fig. 3b, corresponds to the protein region. This compares to a value of around ~2 nm reported in the literature [29]. This discrepancy is assumed to be due to the convolution of the AFM tip.

In the as received enamel the HA crystallites are embedded into the organic matrix and hence the grain boundaries are less visible.

### FTIR Spectroscopy

**Fig. 4:**
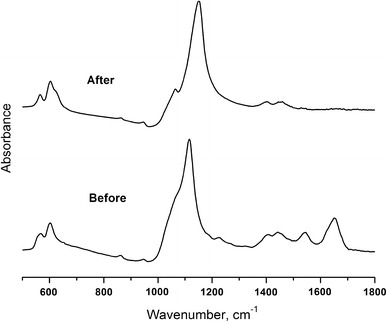
FTIR spectra of dental enamel before and after deproteination

FTIR spectra (500–1,800 cm^−1^) from enamel samples before and after hydrazine treatment are presented in Fig. 4. The large peak around 1,100 cm^−1^ corresponds to the *v*_3_ antisymmetric PO stretching mode and the *v*_1_ symmetric stretching mode of the PO_4_^3−^ group in the HA crystallites [30]. Strong phosphate bands are also seen around 600 cm^−1^. The peak at 870 cm^−1^, seen in both spectra, is attributed to group CO_3_^2−^, found in carbonated hydroxyapatites [31]. The presence of such peaks in the spectra of dental enamel both before and after treatment confirms that the deproteination process does not significantly eliminate these ions from the sample.

The peaks observed around 1,300–1,700 cm^−1^ in the spectrum of the enamel before hydrazine treatment are indicative of the amide bands of the protein component [32], C=O stretching band around 1,650 cm^−1^ (amide I), N–H bending vibration around 1,550 cm^−1^ (amide II) and the amide III band around 1,350 cm^−1^ which correspond to a mixture of coordinated displacements in the amide group. These spectral features are clearly eliminated after the deproteination treatment indicating loss of the organic matrix from the sample.

### Electrostatic Double-Layer

**Fig. 5:**
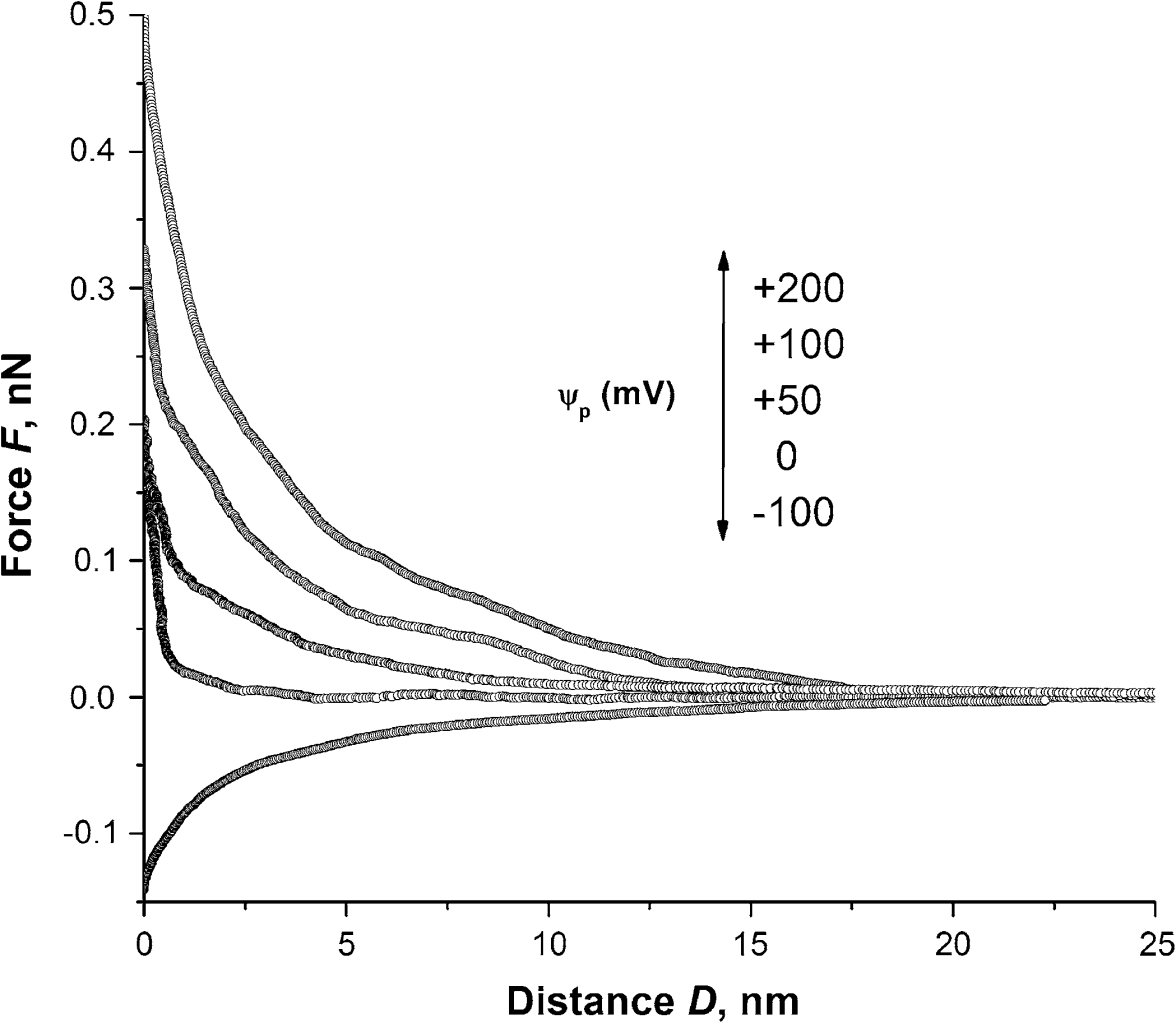
Approaching force curves obtained using various tip voltages on as received tooth enamel surface in SAGF medium with pH 6.8

To find the optimum working potential on the force microscopy tip, the applied voltage was varied incrementally between −100 and +200 mV. Figure 5 shows the force-distance curves between the conductive AFM tip and the as received enamel surface immersed in the SAGF solution at pH 6.8, for various ψ_p_ values. The AFM technique employed here does not have high lateral resolution as the measured charges come from regions well beyond the tip apex radius. This is indeed an advantage as it provides average surface analysis over several HAP crystallites. The enamel-tip interaction is a strong function of the applied potential; as the voltage polarity changed from positive to negative, the force between the tip and surface went from attractive to repulsive. The ability to control tip-sample interaction by varying the tip voltage is evidence for the electrostatic origin of this interaction since other interactions such as van der Waals, hydrophobic and hydration would not display such voltage dependence. Based on these findings, the tip potential ψ_p_ was fixed at a value of −120 mV for subsequent analyses, to maximize the signal to noise while minimizing heating and diffusion effects.

**Fig. 6:**
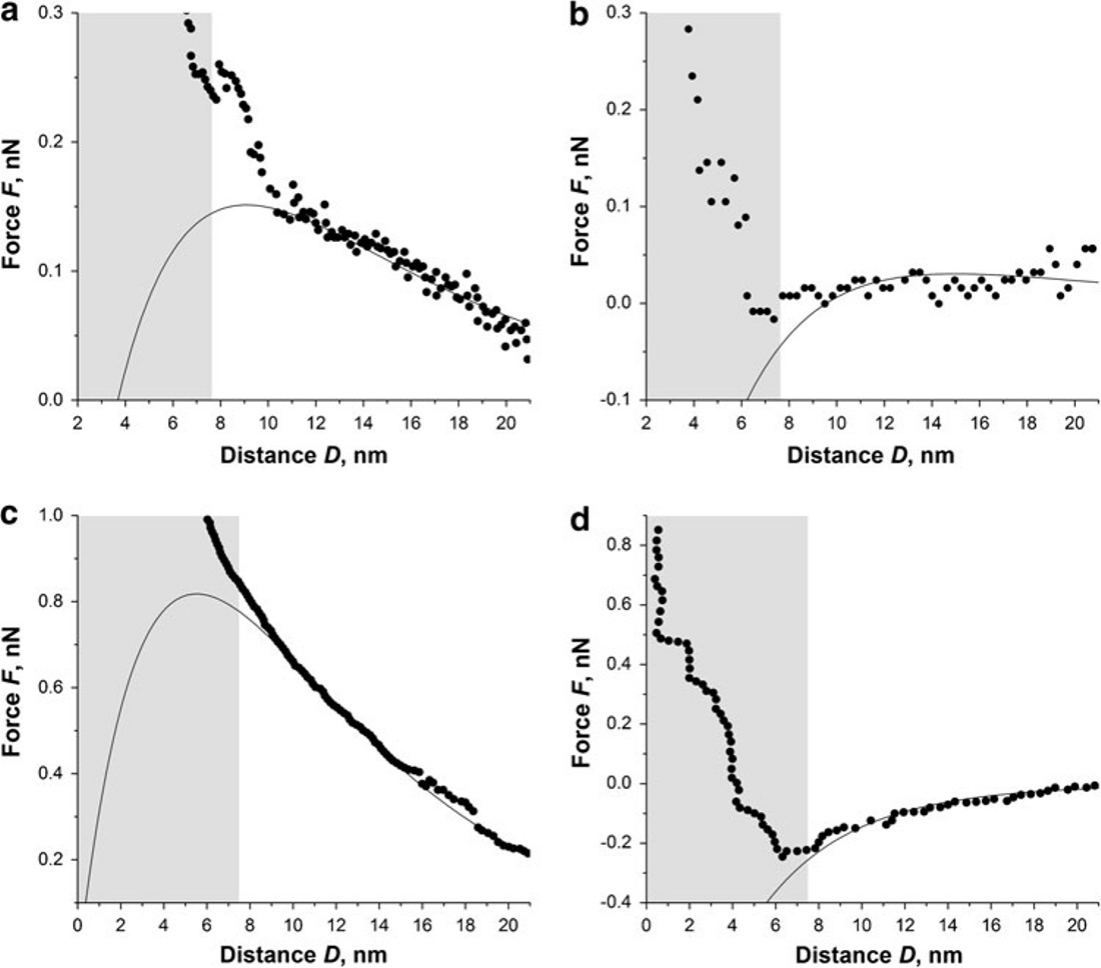
Approaching force curves for as received and deproteinated tooth enamel surfaces immersed in SAGF medium with pH 6.8 (**a**, **b**) and SAGF medium with pH 4.2 (**c**, **d**). *Solid lines* are best fits to the DVLO theory (see Table 2). *Grey areas* show schematically the Debye length calculated for SAGF media

Figure 6 shows the force curves obtained from the two enamel samples (as received and treated) in SAGF solutions with pH 6.8 and 4.2 at ψ_p_ = −120 mV. For the as received sample, the force curves show evidence of electrostatic double-layer interactions. The onset of these interactions, not shown here, is at ~40 nm separations for both pH values. Significant changes in the force curve are observed when the SAG pH is reduced to 4.2 (Fig. 6c); the magnitude of the repulsive force increases. Considering the negative tip potential, this indicates that the enamel surface becomes negatively charged. For the treated sample, the interaction is at a much shorter range, ~8 nm, and is slightly attractive. On lowering the pH, this attractive force increases in magnitude and extends over a larger separation distance. This can be interpreted as the treated sample becoming increasingly positively charged as the environment becomes more acidic.

In continuum theory, the potential distribution due to an electrostatic double layer is determined from the Poisson–Boltzmann equation. The relevant boundary conditions are either constant potential [33] or constant charge [34] on the surface. In practice, selection of appropriate boundary conditions depend on materials used and lies between these extremes [18]. In the present study, a constant potential boundary condition was applied which corresponds to the regulation of *ψ*_*P*_ by the potentiostat. The aim of this modeling is to determine the enamel surface potential *ψ*_*S*_ defined with respect to the bulk of the electrolyte. The DVLO theory, applied to the case of the sphere–plane model (Fig. 2) under the constant potential assumption, computes the interaction force *F* between two surfaces by pair-wise summation as given by Eq. 1, [17, 18]1where *R* is the effective radius of the probe tip, *D* is the separation distance between the tip and the surface, *ε*_0_ is the vacuum permittivity, *ε* is relative dielectric constant of the medium (aqueous medium in these experiments), and *κ* is the inverse of the Debye length in the electrolyte, given by Eq. 2,2where *e* is the electron charge, *c*_*i*_ is the concentration of ions of type *i* in bulk solution, *z*_*i*_ is the charge, *T* is the absolute temperature and *k* is the Boltzmann constant. For aqueous solutions at 25°C and converting ion concentration into molar terms, the Debye parameter yields  where *c*_*i*_ is in M (mol/l) and *κ* is in m^−1^. The Debye length (1/κ) for SAGF solution, calculated from Eq. 2, was found to be 7.6 nm. As the separation distance approaches this value, the surface potential cannot be defined with respect to the bulk of the electrolyte and the model becomes invalid. Indeed, Fig. 5 shows that the data here depart from this model for D values below ~8 nm. Nonetheless, for separation distances greater than the Debye length the model fits the data and can be used to calculate surface charges as per Eq. 3;3

In the present case, the following fixed parameters were used: surface voltage of probe (*ψ*_*P*_ = −120 mV), effective radius of tip (*R* = 58 nm), and *ψ*_*S*_ (mV) as the only free variable fitting parameter. The resulting best fit surface potentials and surface charges are presented in Table 2. For the as received enamel in SAGF solution in both neutral (6.8) and acid (4.2) pH, the values of surface potential are negative. This is in agreement with previously reported results [8]. This suggests a cationic selectivity of intact enamel and a negative fixed charge on the enamel surface. The negative potential present on the as received enamel increased when the pH was lowered. This response to the environment representative of acid attack is the natural protection that occurs through the attraction of positive ions of calcium from saliva and delivering these ions to the enamel layer.

**Table 2:** Electrical surface potentials of tooth enamel surfaces obtained by fitting the experimental force curves to the DVLO theory under constant potential assumption

Sample/medium	Surface potential, (mV)	Surface charge, (C/m^2^)
As received enamel in SAGF, pH 6.8	−70 ± 3	−0.0064
As received enamel in SAGF, pH 4.2	−120 ± 4	−0.0102
Deproteinated enamel in SAGF, pH 6.8	−35 ± 2	−0.0032
Deproteinated enamel in SAGF, pH 4.2	+10 ± 2	0.0009

For the deproteinated sample, the surface potential is less negative in the “physiological” SAGF condition (pH 6.98), it is slightly positive in the acid media condition. The sign and size of surface charges on the deproteinated enamel, at pH 6.8 (−0.0032 C/m^2^) is in good agreement with previously reported values, for example −0.005 to −0.02 C/m^2^ obtained for HA surfaces at physiological relevant conditions [10, 12]. Minor variations in values reported here may be explained by the orientation of HA crystallites in human enamel. Indeed, others have found that the surface charge density varies significantly from −0.0037 to −0.072 C/m^2^ depending on the HA crystal plane [10]. Generally, dental enamel surfaces present the HA lattice hexagonal basal planes [35]. The real situation may be considerably more complex as indicated by an AFM study which has shown that the HA crystals in dental enamel have different charge domains distributed along the HA major axis, perpendicular to the surface [9]. The significance of these results to the present study is that, as enamel is nano-structured and ion-permeable, these different charged layers may contribute to the measured surface potential.

The present results clearly demonstrate that deproteination of the enamel shifts the average surface charge density in the positive direction. Based on a schematic structural model of the embedding of HA nanocrystals within dental enamel [36], we estimate that the protein content represents only ~10% of the enamel surface area. Despite this, it plays a significant role in neutralizing the formation of a positively charged diffuse layer near the tooth surface. In an acid environment, variations between surface charges of as received and deproteinated enamel becomes even more pronounced.

## Summary and Conclusions

The direct measurement of surface charge density of dental enamel has been reported for the first time using double layer force spectroscopy. Experimental force-distance curves have been fitted using DLVO theory.

FTIR spectroscopy and AFM topography characterization were used for the analysis of dental enamel samples before and after deproteinization. The results show that the treatment with hydrazine hydrate does not alter enamel mineral composition or morphology, but effectively eliminates the dental proteins and organic matrix. A number of very small cracks are observed on the deproteinated enamel surface suggesting that the protein component may also have a role to play in determining the mechanical properties of enamel.

By comparing the charge on sound and deproteinated enamel surfaces when in contact with aqueous environments at different pH, we have shown that the organic matrix of dental enamel plays a significant role in regulation of electrostatic properties of human tooth and, as a result, contributes to the ability of dental enamel to defend against an acidic attack.
